# Exendin-4 Protects Mitochondria from Reactive Oxygen Species Induced Apoptosis in Pancreatic Beta Cells

**DOI:** 10.1371/journal.pone.0076172

**Published:** 2013-10-24

**Authors:** Zhen Li, Zhiguang Zhou, Gan Huang, Fang Hu, Yufei Xiang, Lining He

**Affiliations:** Diabetes Center, Second Xiangya Hospital, and Institute of Metabolism and Endocrinology, Key Laboratory of Diabetes Immunology, Ministry of Education, Central South University, Changsha, China; University of Hong Kong, China

## Abstract

**Objective:**

Mitochondrial oxidative stress is the basis for pancreatic β-cell apoptosis and a common pathway for numerous types of damage, including glucotoxicity and lipotoxicity. We cultivated mice pancreatic β-cell tumor Min6 cell lines *in vitro* and observed pancreatic β-cell apoptosis and changes in mitochondrial function before and after the addition of Exendin-4. Based on these observations, we discuss the protective role of Exendin-4 against mitochondrial oxidative damage and its relationship with Ca^2+^-independent phospholipase A2.

**Methods:**

We established a pancreatic β-cell oxidative stress damage model using Min6 cell lines cultured *in vitro* with tert-buty1 hydroperoxide and hydrogen peroxide. We then added Exendin-4 to observe changes in the rate of cell apoptosis (Annexin-V-FITC-PI staining flow cytometry and DNA ladder). We detected the activity of the caspase 3 and 8 apoptotic factors, measured the mitochondrial membrane potential losses and reactive oxygen species production levels, and detected the expression of cytochrome c and Smac/DLAMO in the cytosol and mitochondria, mitochondrial Ca_2_-independent phospholipase A2 and Ca^2+^-independent phospholipase A2 mRNA.

**Results:**

The time-concentration curve showed that different percentages of apoptosis occurred at different time-concentrations in tert-buty1 hydroperoxide- and hydrogen peroxide-induced Min6 cells. Incubation with 100 µmol/l of Exendin-4 for 48 hours reduced the Min6 cell apoptosis rate (*p*<0.05). The mitochondrial membrane potential loss and total reactive oxygen species levels decreased (*p*<0.05), and the release of cytochrome c and Smac/DLAMO from the mitochondria was reduced. The study also showed that Ca^2+^-independent phospholipase A2 activity was positively related to Exendin-4 activity.

**Conclusion:**

Exendin-4 reduces Min6 cell oxidative damage and the cell apoptosis rate, which may be related to Ca^2^-independent phospholipase A2.

## Introduction

Changes in mitochondrial function and structure constitute the basis of pancreatic β-cell apoptosis and are a fundamental cause of insulin resistance. The long-term metabolic load, including hyperglycemia and hyperlipidemia, causes pancreatic β-cell mitochondrial superoxide (ROS) to accumulate and the mitochondria to enlarge. Inner membrane pores are formed, and cytochrome c is released from mitochondrial inner membranes into the cytoplasm. This release activates a series of apoptosis factors, including caspase 3, 6, 7, 8 and 9, and results in DNA fragmentation, protein denaturation, membrane phospholipid oxidation and increased apoptosis. Simultaneously, because of the mitochondrial fatty acid oxidation barrier and increased intracellular acyl-coenzyme A and acylglycerol levels, an insulin-mediated glucose transport key signal is activated and glucose transport is blocked, which leads to insulin resistance [1]–[5].

Cardiolipin is a major component of the mitochondrial inner membrane. Cardiolipin possesses a unique dimer structure with two negative charges and four fatty acyl groups that easily combine with ROS and produce an oxidative reaction; therefore, cardiolipin is a target of oxidative damage [6]–[8]. Ca^2+^-independent Phospholipase A2 (iPLA-2) is located within the mitochondrial inner membrane and is an important substance for remodeling and damage repair and for assisting and accelerating apoptosis and the clearance of damaged cells [9]–[15].

Exendin-4 is an analog of glucagon-like peptide-1(GLP-1). It promotes insulin gene transcription, synthesis and secretion; inhibits the apoptosis of pancreatic β-cells at the gene level; and promotes proliferation and regeneration. Currently, Exendin-4 is a relatively common anti-diabetic drug in application and research. Exendin-4 is also the only drug that can theoretically delay and reverse the development of diabetes [16]–[22].

In the present study, mice pancreatic β-cell tumor Min6 cell lines were cultured *in vitro*, and pancreatic β-cell apoptosis and changes in mitochondrial function were observed before and after the application of Exendin-4. In this paper, the protective role of Exendin-4 in the diabetic mitochondrial function barrier and the relationship between this role and iPLA-2 are discussed.

## Research Methods

We used mice pancreatic β-cell tumor Min6 cell lines (the primary culture cell came from the Shanghai Institute of Biological Products and passed to 30 generations), high-glucose medium (DMEM) and 15% fetal calf serum. We performed *in vitro* cultivation at 37°C and 5% CO_2_ and used different concentrations of tert-buty1 hydroperoxide (t-BHP) and H_2_O_2_ to establish a cell oxidative damage model.

### Groups

Different intervention groups were established based on time-concentrations. The t-BHP groups were divided into the following subgroups based on intervention time: 30, 45 and 60 min and 4, 8, 12 and 24 hours. The intervention concentrations were 0, 50, 100, 125, 200 and 400 µmol/l. The H_2_O_2_ group intervention times were 4, 8, 12 and 24 hours with concentrations of 0, 50, 100 and 200 µmol/l.

### Determination of the apoptosis rate

Two methods were adopted to detect the cell apoptosis rate and determine the best intervention time and concentration.

Initially, Annexin-V-FITC-PI apoptosis detection assay kits (Sigma, Saint Louis, Missouri, USA) were used to confirm the best intervention time-concentration. Annexin V is a sensitivity index used to detect early cell apoptosis. Propidium iodide (PI) permeates the cell membrane and dyes cell nuclei red during the middle and later stages of apoptosis and in dead cells, thus distinguishing cells at different apoptotic stages.

We inoculated Min6 cells in a six-well cell culture plate; each well contained approximately 1×10^6^ cells. The groups were divided based on time-concentration. After each reaction with each tBHP concentration was allowed to proceed for the allotted time, the cells were harvested, counted and washed with cold phosphate buffered saline (PBS) and digested with pancreatic enzymes. Annexin-V-FITC and PI were used for staining following the manufacturer's staining procedure (SIGMA Annexin V-FITC Apoptosis Detection Kit). Flow cytometry was used for detection (Becton Dickinson, FACScan), and Cell Quest TM software was used to analyze the results. On the scatter chart of the dual-variable flow cytometry, the lower left quadrant displayed living cells (FITC−/PI−), the upper left quadrant displayed necrotic cells (FITC−/PI+), the right upper quadrant displayed late-stage apoptotic cells (FITC+/PI+) and the right lower quadrant displayed early-stage apoptotic cells (FITC+/PI−). Simultaneously, four comparison groups were established: a blank control, which contained normal cells without dyes or treatments; normal cells with AV-FITC (used for the horizontal axis to confirm and distinguish the four quadrants); normal cells with PI (used to confirm the vertical axis); and normal cells with both dyes added. The experiment was repeated three times.

### DNA fragment analysis

The most prominent feature and biochemical characteristic of cell apoptosis is the degradation of DNA into oligonucleotide fragments, which are composed of approximately 180–200 bp, or DNA polymers, and agarose gel electrophoresis reveals a characteristic ladder-shaped belt. Based on the biochemical characteristics of cell apoptosis described above, an Apoptotic DNA Ladder Kit (Roche Applied Science, Mannheim, Germany) was used to detect apoptosis.

Each well was inoculated with 2×10^6^ Min6 cells washed with cold PBS, digested with 0.25% pancreatic enzymes for 1 min and repeatedly blown with cold PBS. The cells were centrifuged twice at 200 g for 5 min; then, 200 µl of binding buffer was added, and the cells were incubated at room temperature (15–25°C) for 10 min. Altogether, 100 µl of isopropyl alcohol was added, and the cells were centrifuged twice at 8,000 g for 1 min. The cells were repeatedly centrifuged at 13,000 g for 1 min and placed in a clean 1.5-ml centrifuge tube before 200 µl of eluent (preheated to 70°C) was added. The cells were kept at room temperature for 5 min and were then centrifuged at 8,000 g for 1 min to obtain the target DNA. After staining with ethidium bromide, 1% agarose gel electrophoresis and pulsed-field gel electrophoresis were used to analyze the results.

### Detection of changes in the cell lipid peroxide content

During oxidative stress, a portion of the intracellular fatty acids is oxidized into a series of complex compounds, including malondialdehyde (MDA). The level of lipid oxidation can be detected by determining the MDA level. This study used an Oxltek TBARS assay kit (Zeptometrix Corporation, NY, USA). Using the colorimetric method, we performed quantitative MDA detection. First, we used 100 µl of cold PBS to suspend the cells, and then we added 100 µl of sodium dodecyl sulfate (SDS) solution. After vortex mixing, 500 µl of thiobarbituric acid (TBA)/buffer reagent (acetic acid+sodium hydroxide+TBA solution) was added. The solution was incubated at 95°C for 60 min and then chilled on ice for 10 min. The supernatant was then transferred to a 96-well plate, and an ELISA reader was used to determine the absorbance and concentration. The excitation wavelength was established at 535 nm, and the maximum emission wavelength was 553 nm.

### Detection of the activity of caspase 3 and 8 apoptosis factors

A caspase 3 and 8 detection assay kit (Beyotime Institute of Biotechnology, Haimen, Jiangsu, China) was used to detect caspase activity. Caspase 3-mediated protein shear is the key enzyme in cell apoptosis, chromatin-solid contraction, DNA fragmentation and cell foaming. Caspase 3 and 8 catalyze Ac-DEVD-pNA (acetyl-Asp-Glu-Val-Asp p-nitroanilide) and Ac-IETD-pNA (acetyl-Ile-Glu-Thr-Asp-p-nitroanilide), respectively, were used to produce yellow pNA (p-nitroaniline). We detected caspase 3 and 8 activity by measuring absorbance.

Each well was inoculated with 1×10^6^ Min6 cells, and 60 µl of lysis buffer was used to lyse the cells on ice for 15 min. The cells were centrifuged at high speed, and the supernatant was collected. The standard product was diluted to concentrations of 0, 10, 20, 50, 100 and 200 µM. Various reactants were added according to the assay kit instructions. After mixing, 10 µl of Ac-DEVD-pNA or Ac-IETD-pNA (2 mM) was added. The cells were incubated at 37°C for 60 to 120 min. A405 was determined when a color change occurred. The caspase 3 (or 8) enzyme activity unit in each unit weight of protein was calculated as follows: optical density (OD) value/protein concentration. The experiment was repeated three times.

### Detection of apoptosis-related protein expression

We isolated the mitochondria and cytoplasm according to the Cell Mitochondria Isolation Kit manual (Beyotime Institute of Biotechnology, Haimen, Jiangsu, China). Cold PBS was used for washing, and the Min6 cells were collected. The number of cells in each sample was greater than 2×10^6^. The cells were centrifuged at 200 g for 5 min and then 100 µl of mitochondria isolation reagent containing phenylmethanesulfonyl fluoride (PMSF) was added. After suspension, the cells were placed in an ice bath for 10 to 15 min and then transferred to a glass homogenizer for homogenization. Subsequently, 30–50 µl of Trypan blue staining solution was added to determine the percentage of positive (blue) cells. When the percentage was greater than 50%, the cells were centrifuged at 600 g at 4°C for 10 min. The supernatant was then transferred to another centrifuge tube and centrifuged at 11,000 g at 4°C for 10 min. The supernatant and precipitate were collected. The precipitates contained the isolated cell mitochondria. The supernatant was centrifuged at 12,000 g at 4°C for 10 min to obtain the cytoplasm proteins without mitochondria. Finally, we used Western blotting, Anti-iPLA2, Anti-cPLA2, Anti-Cytochrome C, and Anti-Smac/DIABLO (Milipore, Temecula, California, USA) to detect the expression levels of the corresponding proteins.

### Detection of changes in the mitochondrial membrane potential

A reduction in the mitochondrial membrane potential is the landmark event of early cell apoptosis. When the mitochondrial membrane potential (ΔΨm) was relatively high, JC-1 accumulated in the matrix of the mitochondria to form polymers and fluoresced red. When ΔΨm was relatively low, the monomer fluoresced green. The JC-1 monomer's maximum activation wavelength is 514 nm, and the maximum emission wavelength is 529 nm. The JC-1 polymer's maximum excitation wavelength is 585 nm, and the maximum emission wavelength is 590 nm.

As described in the JC1-Mitochondrial membrane potential assay kit (Abcam, Hongkong, China) manual, the pretreated cells were collected, centrifuged at 200 g for 5 min and washed twice with ice-cold PBS; then, 100 µl of JC-1 (10 µmol/l) was added. The cells were incubated at 37°C for 30 min and centrifuged at 200 g for 5 min. They were then washed once with cold PBS. After 100 µl of cold PBS was added to generate a suspension, the cells were transferred to a 96-well plate. The ELx800 Absorbance Microplate Reader (Bio-Tek Instruments, INC. Vermont, USA) was used to read the fluorescence, and the ratio of green to red fluorescence was calculated. An increase in the proportion of green fluorescence indicated that the percentage of apoptosis was relatively high.

### Detection of changes in the mitochondrial ROS content

Dihydroethidium is the most common superoxide anion fluorescence probe. After consumption by living cells, superoxide anions dehydrogenate dihydroethidium in the cell and produce ethidium. Dihydroethidium exhibits blue fluorescence with a maximum excitation wavelength of 370 nm and a maximum emission wavelength of 420 nm. After dehydrogenation, dihydroethidium combines with RNA or DNA to fluoresce red with a maximum excitation wavelength of 300 nm and a maximum emission wavelength of 610 nm. Initially, dihydroethidium (Sigma, Missouri, USA) was diluted in DMSO to prepare a 5000-µmol/l mother solution. Before detection, the mother solution was diluted to 2 µmol/L and incubated with the pretreated cells at 37°C for 15 min. The solution was then centrifuged at 200 g for 5 min, washed once with cold PBS and transferred to a 96-well plate. The ELx800 Absorbance Microplate Reader (Bio-Tek Instruments, INC. Vermont, USA) was used to read the fluorescence and detect the fluorescence ratio of blue to red. An increase in the proportion of red fluorescence indicated that the percentage of apoptosis was relatively high.

### Determination of protein concentration

Under alkaline conditions, the protein reduced Cu^2+^ to Cu^+^. Cu^+^ and a bicinchoninic acid (BCA) reagent formed a purple complex, and its absorbance value was measured at 562 nm. The absorbance values were then compared to the standard curve to calculate the protein concentration. Pierce BCA protein assay kits (Thermo, Rockford, IL, USA) were used.

### Determination of iPLA2 activity

We used a cPLA2 (Ca^2+^-dependent Phospholipase A2) assay kit (Cayman Chemical Company, MI, USA). cPLA2 specifically hydrolyzes arachidonic acid (sn-2 arachidonoyl thioester and thiol release). Thiol can be detected by DTNB (5,5′-dithiobis(2-nitrobenzoic acid)), and the result is equal to the total PLA2 activity. The iPLA2 blocking agent bromoenol lactone (BEL) was then added to calculate the iPLA2 content of the total PLA2 and iPLA2 activity.

### Extraction of iPLA2 RNA for further analysis by RT-PCR

RNA extraction was based on the method provided by TRIZOL Reagent (Invitrogen, Florida, USA), which is divided into six steps: homogenization, phase separation, RNA precipitation, RNA washing, dissolution and RNA determination. For reverse-transcription PCR, an ImProm-II™ reverse transcription assay kit (Promega, Wisconsin, USA) was used. Based on the method provided by Promega, a 20-µl reaction volume was used to reverse-transcribe 1 µg of total RNA. This procedure was divided into two steps: RNA combination with a primer and denaturation and the reverse-transcription reaction. The iPLA2 primer sequences were designed using Primer 3 software, and the target sequences were downloaded from the GenBank database.

Sense strand: (5′- GCCCTGGCCATTCTACACAGTA-3′)

Anti-sense strand: (5′- CACCTCATCCTTCATACGGAAGT-3′)

18S sense strand: (5′- GCCGCTAGAGGTGAAATTCTTG-3′)

Anti-sense strand: (5′- CATTCTTGGCAAATGCTTTCG-3′).

SYBRGreen was used for one-step real-time quantitative PCR analysis. The internal reference gene was 18S. The analysis was divided into two steps: preparation of the reaction system and real-time quantitative PCR analysis.

### Exendin-4 intervention

Exendin-4 (Taili Biological Engineering Co., Ltd. Dongguan, Guangdong, China) was added based on the induction of apoptosis at an early stage. A group intervention was performed based on time-concentration.

The following groups were used: 1) control group; 2) t-BHP group (25, 50, 100, 125, 200 and 400 µmol/l); 3) H_2_O_2_ group (50, 100 and 200 µmol/l); 4) Exendin-4 (10 and 100 nmol/l); 5) Min6 cells pretreated with Exendin-4 for 24 hours and then added to t-BHP (200 and 400 µmol/l); 6) Min6 cells pretreated with Exendin-4 for 24 hours and then added to H_2_O_2_ (50, 100 and 200 µmol/l); 7) Min6 cells pretreated with Exendin-4 for 48 hours and then added to t-BHP (200 and 400 µmol/l); and 8) Min6 cells pretreated with Exendin-4 for 48 hours and then added to H_2_O_2_ (50, 100 and 200 µmol/l).

When the cells were harvested, the occurrence of apoptosis, changes in mitochondrial membrane potential and mitochondrial generation of ROS, caspase 3 and caspase 8 activities, protein cytochrome *c* and Smac/DIABLO expression in mitochondria and cytoplasm, and iPLA2 expression and activity were evaluated.

### Statistical analysis

SPSS 16.0 was used for the statistical analyses. The data were expressed as the means±S.D. The data were analyzed using a general linear model and repeated measures analysis of variance. *p*<0.05 was considered statistically significant.

## Results

### Optimal time-concentration intervention (200 µmol/L of t-BHP for 24 hours)

**Figure 1:**
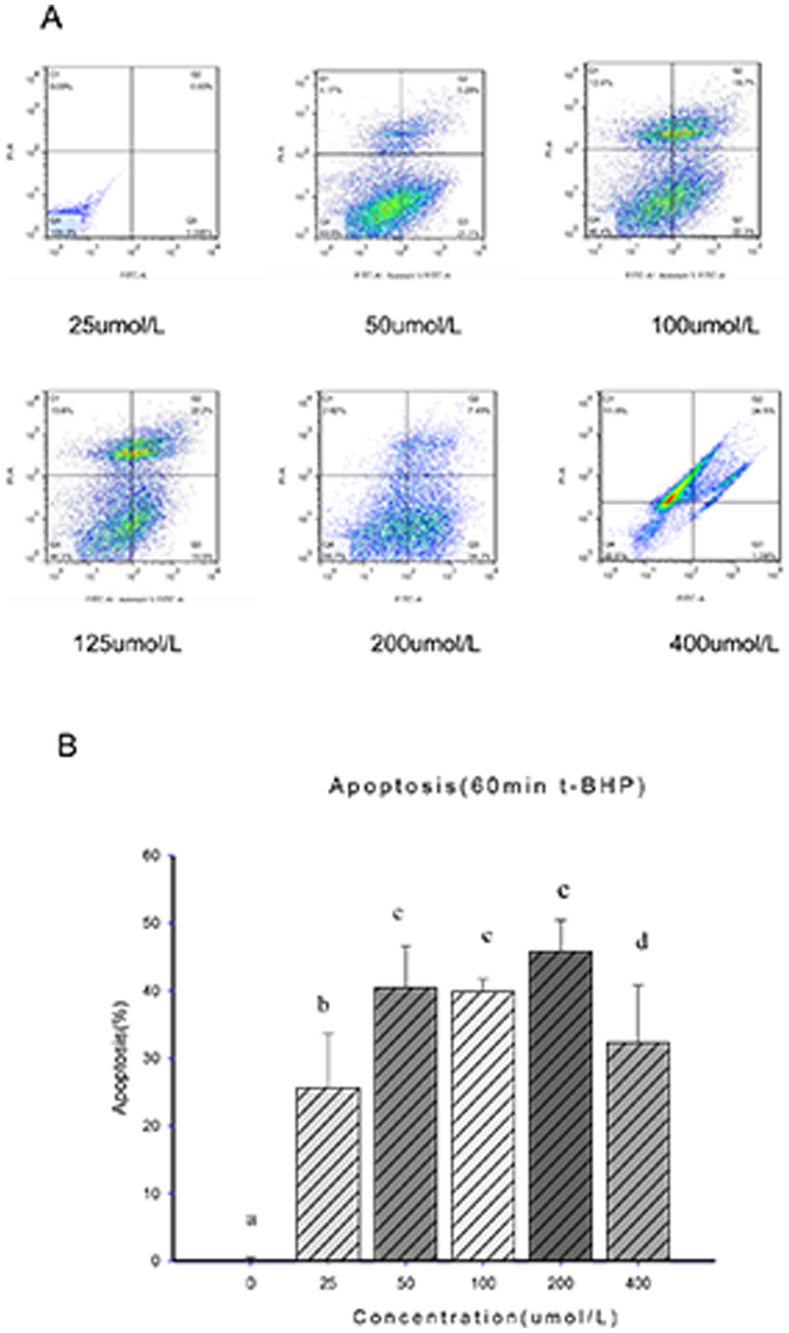
Determination of the apoptosis rate in 1 A, FACS analysis of apoptosis. The different concentrations of t-BHP were allowed to react for 1 hour to induce early (Annexin V-positive and PI-negative) and late apoptosis (Annexin V- and PI-positive) in Min6 cells, and then, FACS analysis was performed. Figure B summarizes the results and includes a comparison of the different t-BHP concentrations and Min6 cell apoptosis percentages of early-stage and late-stage. The data are expressed as the means±S.D (*n = *6). a, b, c and d show comparisons between the blank control and different concentrations of t-BHP (*p*<0.05).

**Figure 2:**
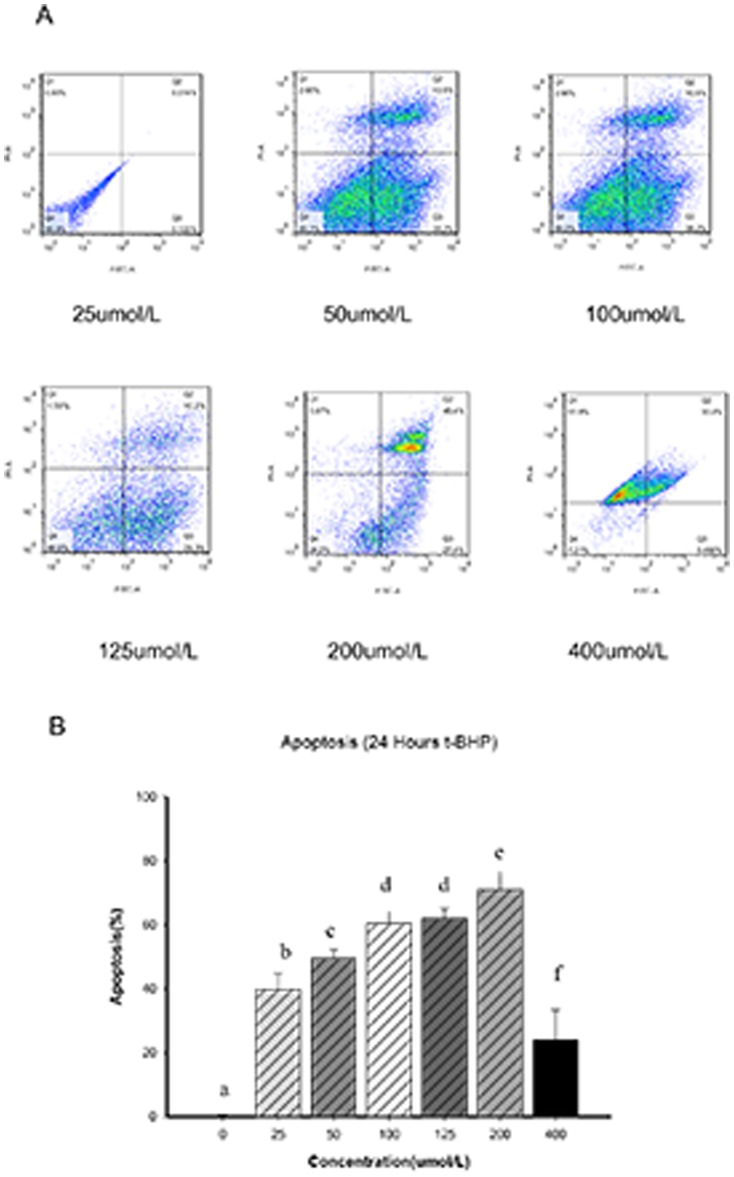
Determination of the apoptosis rate in 24 A, FACS analysis of apoptosis. The different concentrations of t-BHP were allowed to react for 24 hour to induce early (Annexin V-positive and PI-negative) and late apoptosis (Annexin V- and PI-positive) in Min6 cells, and then, FACS analysis was performed. Figure B summarizes the results and includes a comparison of the different t-BHP concentrations and Min6 cell apoptosis percentages of early-stage and late-stage. The data are expressed as the means±S.D (*n = *6). a, b, c, d, e and f show comparisons between the blank control and different concentrations of t-BHP (*p*<0.05).

Flow cytometry was used to analyze the concentration/time gradient. The results showed that as the time and concentration increased, the rate of Min6 cell apoptosis gradually increased. These results were shown as two peaks on the concentration/time curve, representing a t-BHP concentration of 200 µmol/l for 1 and 24 hours. At the t-BHP concentration of 200 µmol/l for 1 hour, there was significant early- and late-stage apoptosis. The total rate of apoptosis ranged from 40.1 to 60%, the average early apoptosis rate was 31.55%, the late apoptosis rate was 8.46%, and the average cell mortality was 26.8%. However, as the time increased, the rate of increased apoptosis slowed. At 8 hours, the apoptosis percentage was not significantly different from that at 1 hour (*p*>0.05). At 200 µmol/l of t-BHP for 24 hours, the late-stage apoptosis rate was 46.1%, the early-stage apoptosis rate was 21.6%, and cell mortality was 4.86%. When the concentration reached 400 µmol/l in 1 hour, there was significant apoptosis in the Min6 cells, with more than 90% mortality (Figures 1 & 2).

### Exendin-4 (100 µM for 48 hours) protects cells from mitochondrial stress–induced apoptosis

**Figure 3:**
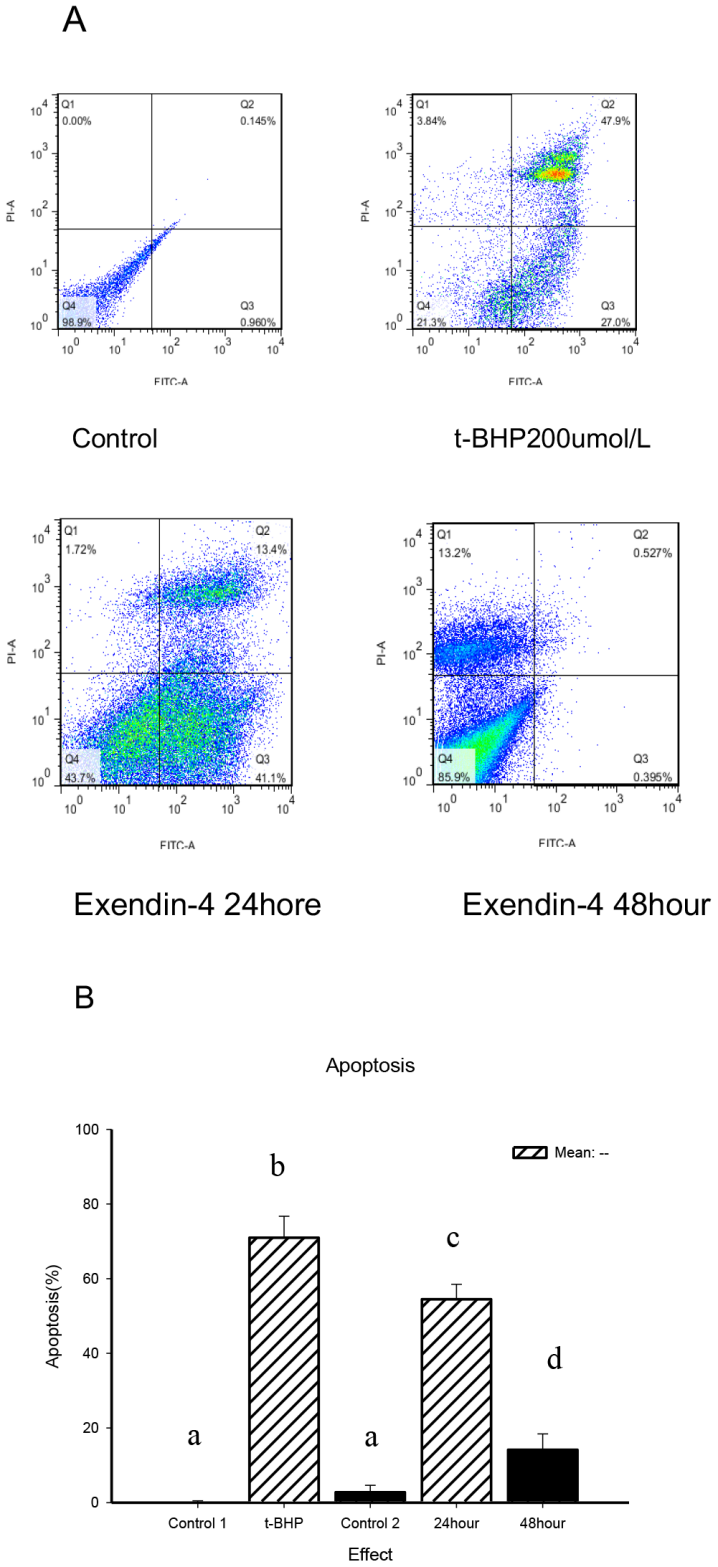
Exendin-4 prevents apoptosis induced by t-BHP. A, FACS analysis of apoptosis. Min6 cells were divided into four groups: control, t-BHP (200 µM for 24 hours), Exendin-4 (100 µM for 24 hours) and Exendin-4 (100 µM for 48 hours). Before being treated with t-BHP, the cells were treated with Exendin-4 for 24 or 48 hours). The cells were collected and stained with Annexin V and PI and analyzed by FACS. Figure B presents a summary of the results and includes a comparison of the apoptosis percentages of early-stage and late-stage. The data are expressed as the means±S.D. (*n* = 6). a, b, c and d indicate the blank control, t-BHP (200 µM for 24 hours), Exendin-4 (100 µM for 24 hours) and group comparisons with the Exendin-4 (100 µM for 48 hours) group (*p*<0.05).

The results of incubating Min6 cells with Exendin-4 showed that as the time increased, the rate of apoptosis decreased (*p*<0.05). Compared with the control group, when Exendin-4 was incubated with Min6 cells for 24 hours and 200 µM t-BHP was added for 24 hours, the total apoptosis rate of the Min6 cells was reduced from 66.7 to 54.5%. When Exendin-4 was incubated with Min6 cells for 48 hours and 200 µM t-BHP was added for 24 hours, the total percentage of dead and apoptotic Min6 cells was reduced to 14.1% (Figure 3).

**Figure 4:**
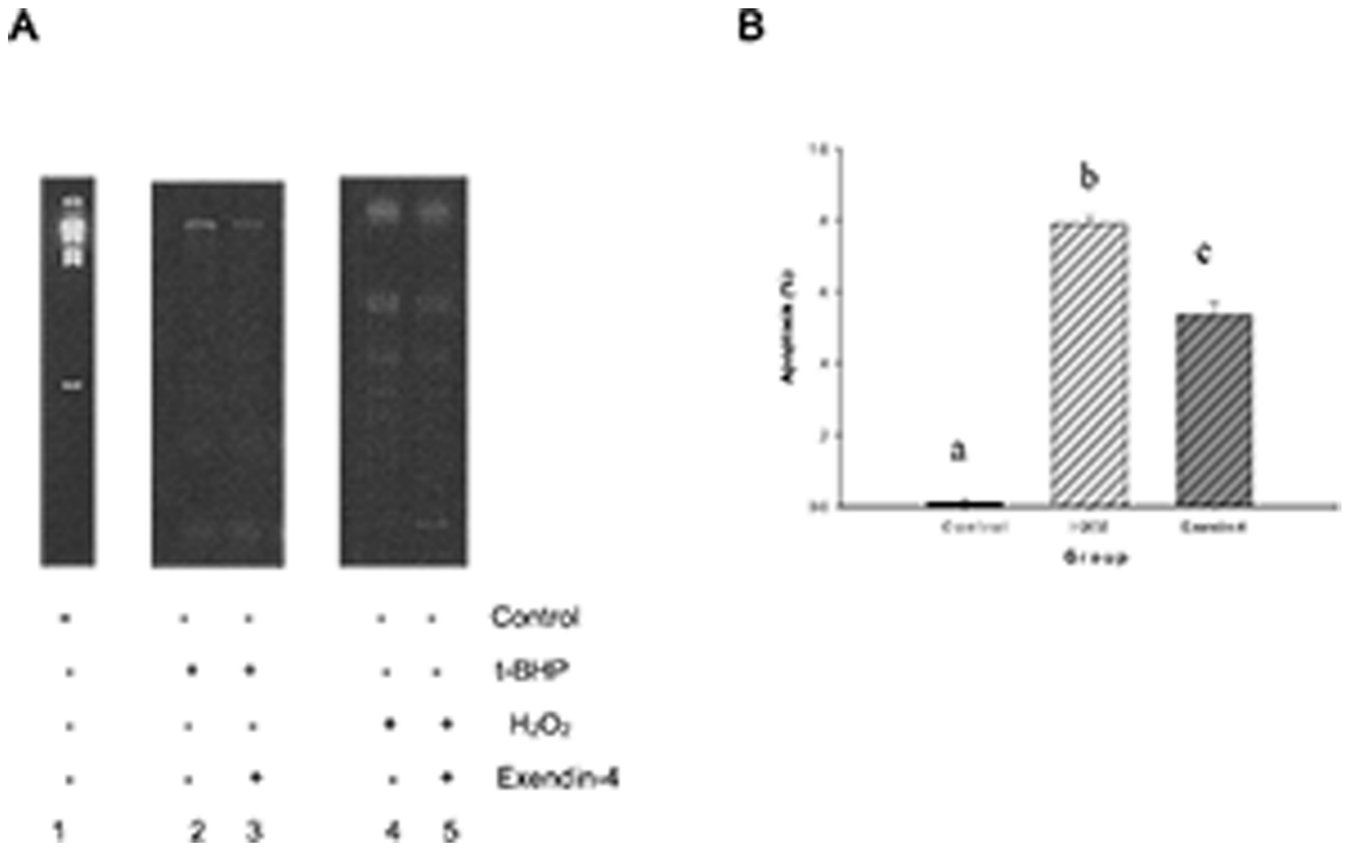
Exendin-4 reduced H_2_O_2_-induced Min6 cell DNA fragmentation. A, Apoptotic DNA ladder analysis. fter t-BHP (200 µM for 24 hours, as in Figure A2) and H_2_O_2_ (100 µM for 24 hours, as in Figure A4) treatment, the DNA was purified using an apoptosis DNA ladder kit and analyzed on agarose gel. Figures A3 and A5 represent Exendin-4 groups that were treated with t-BHP or H_2_O_2_ after treatment with Exendin-4 100 µM for 48 hours. Figure B is a summary of the results and includes the gray comparison among the control group, the H_2_O_2_ (100 µM for 24 hours) group and the Exendin-4 group. The data are expressed as the means±S.D. (*n* = 6). a, b and c indicate the respective comparisons (*p*<0.05).

Using a DNA ladder assay kit, we performed a DNA fragmentation analysis according to the manufacturer's instructions. Initially, at 200 µmol/l of t-BHP for 24 hours, no typical changes in DNA fragmentation were observed. We then used 100 µmol/l of H_2_O_2_ for 24 hours. We applied an orthogonal alternating-pulse electric field in the agarose gel, and the results showed that the DNA exhibited changed fragmentation behavior. We used flow cytometry to detect the percentage of cell apoptosis. The percentage of 100-µM-H_2_O_2_-induced Min6 cell apoptosis was 79.2%. When Exendin-4 was incubated with Min6 cells for 48 hours and 100 µM H_2_O_2_ was added for 24 hours, the DNA fragmentation was attenuated, and the total percentage of dead and apoptotic Min6 cells was reduced to 56.8% (Figure 4).

**Figure 5:**
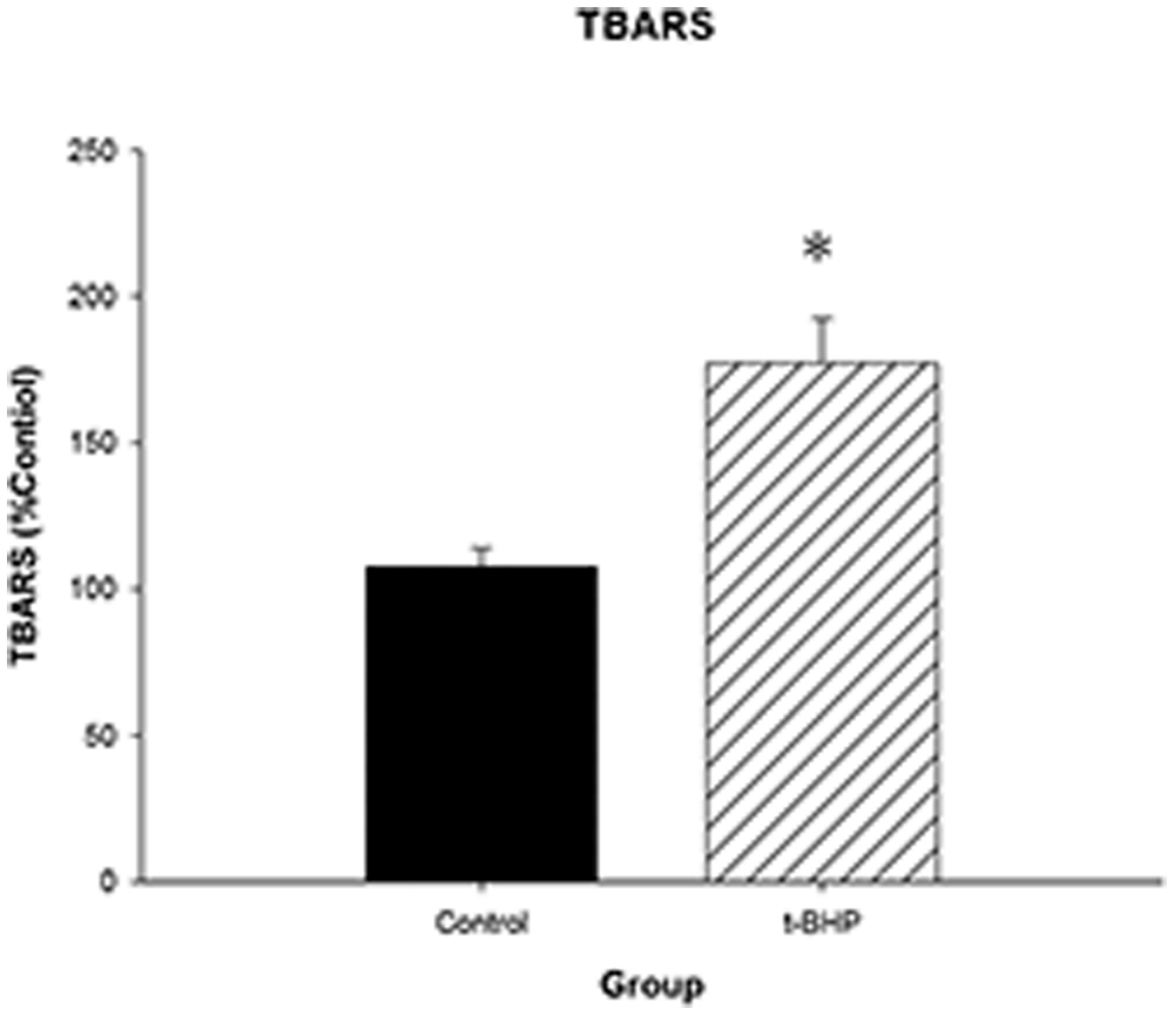
Intracellular oxidative fatty acid content analysis. Cells were collected, treated with trichloroacetic acid/TBARS reagent and incubated at 95°C for 60 min. The supernatants were analyzed, and the values are expressed as percentage of control values. The data are expressed as the means±S.D. (*n* = 6). * indicates a comparison with the blank control group (p<0.05).

An Oxltek TBARS assay kit was used to detect changes in the Min6 cell oxidative fatty acid content. The results showed that as the rate of cell apoptosis increased, the cell oxidative fatty acid content also increased in the t-BHP 100 µM 24-hour group (Figure 5). t-BHP induces an increase in the Min6 cell oxidative fatty acid content.

### Exendin-4 (100 µM for 48 hours) prevents the loss of mitochondrial membrane potential

**Figure 6:**
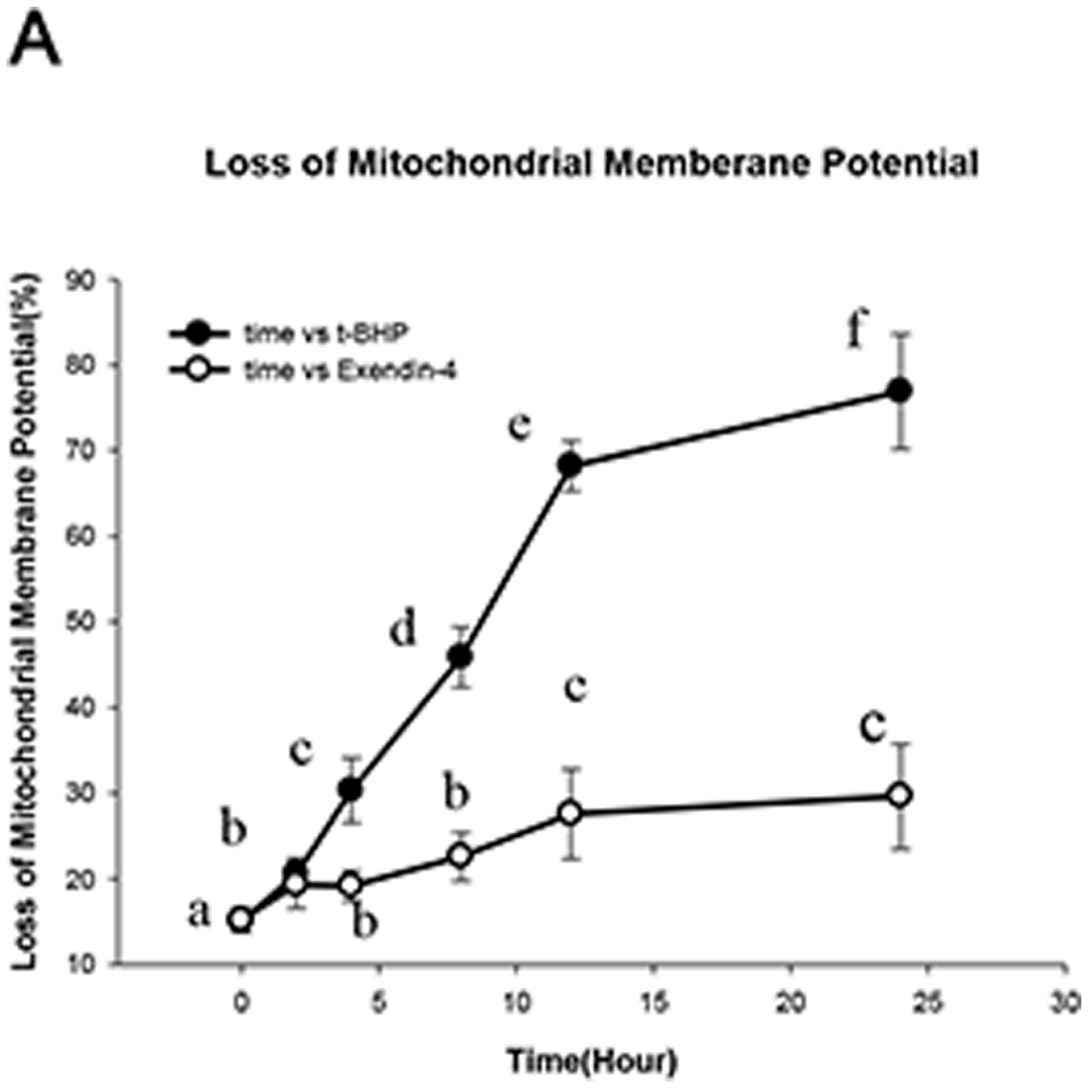
Exendin-4 prevents the loss of mitochondrial membrane potentials. An absorbance microplate reader was used to analyze the loss of mitochondrial membrane potentials. The figure shows the time course of the t-BHP-induced loss of mitochondrial membrane potential. The data are expressed as the means±S.D. (*n = *6). a, b, c, d, e and f indicate the t-BHP groups and Exendin-4 groups at different intervention times (*p*<0.05).

Alterations in the mitochondrial membrane potential represent an early transition in the induction of apoptosis. To determine whether Exendin-4 can prevent the loss of mitochondrial membrane potential, we found that at 100 µmol/l of t-BHP for 2 hours, the mitochondrial membrane potential began to decrease; at 24 hours, the potential loss was approximately 80%. However, after treatment with Exendin-4 (100 µM for 48 hours), the t-BHP induced membrane potential loss decreased (*n* = 6; *p*<0.05; details shown in Figure 6).

### Exendin-4 (100 µM for 48 hours) protects cells from producing more ROS in response to t-BHP treatment

**Figure 7:**
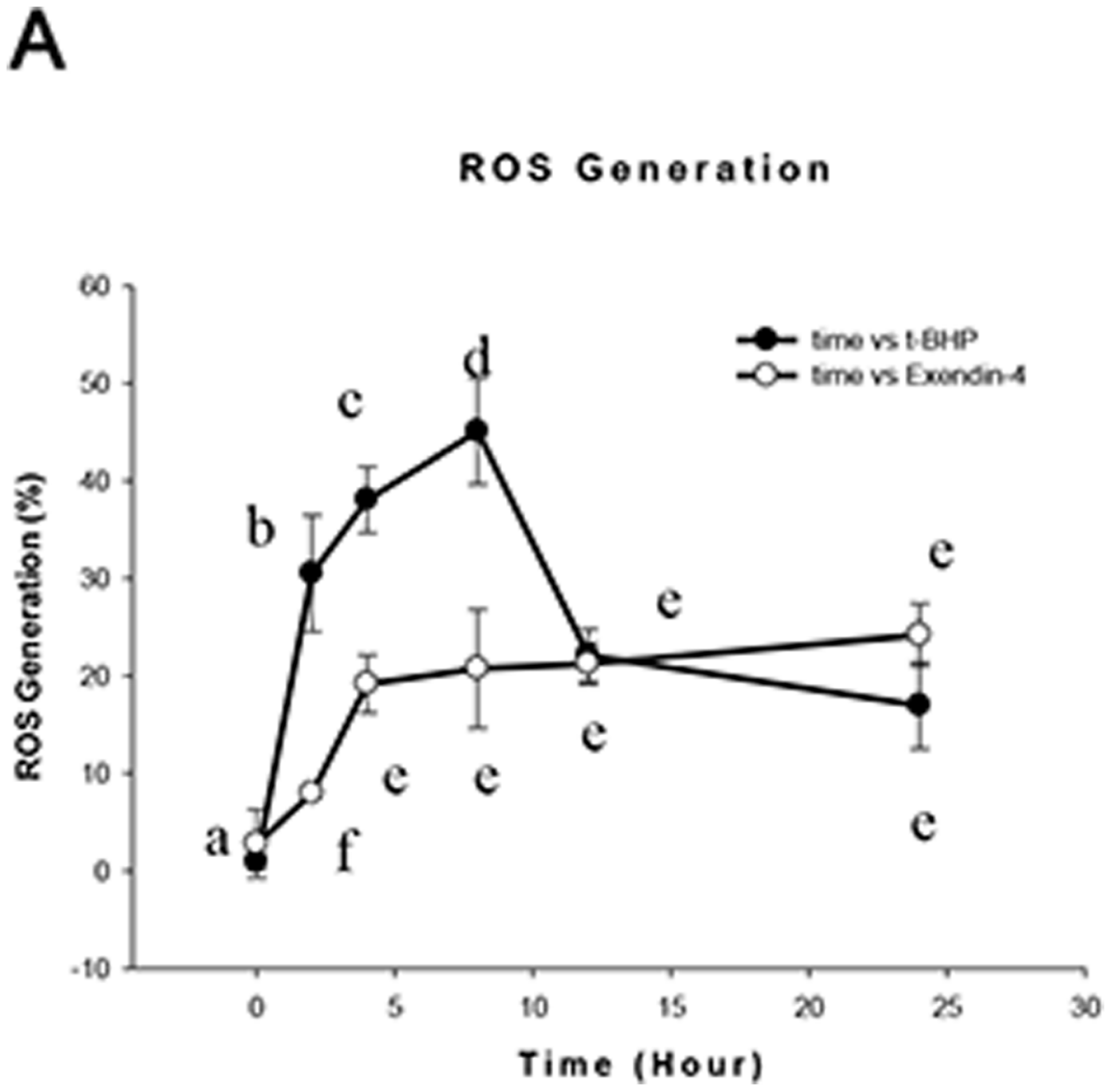
Exendin-4 reduces t-BHP-induced ROS production. Cells were treated with or without Exendin-4 and then with t-BHP for different intervention times, collected, stained with 2 µM HE and analyzed with an absorbance microplate reader. Values are expressed as percentages of total cell counts. The data are expressed as the means±S.D. (*n = *6). a, b, c, d and e indicate the t-BHP groups and Exendin-4 groups at different intervention times (*p*<0.05).

We examined whether Exendin-4 affects the amount of ROS generation by mitochondria response to t-BHP. We found that after treatment with 100 µmol/l of t-BHP for 2 hours, the superoxide content of the Min6 cell mitochondria increased and peaked after 8 hours. With the addition of Exendin-4 (100 µM for 48 hours), the t-BHP-induced ROS generation decreased (*n* = 6; *p*<0.05; details shown in Figure 7).

### Exendin-4 attenuates the release of cell apoptotic proteins from mitochondria

**Figure 8:**
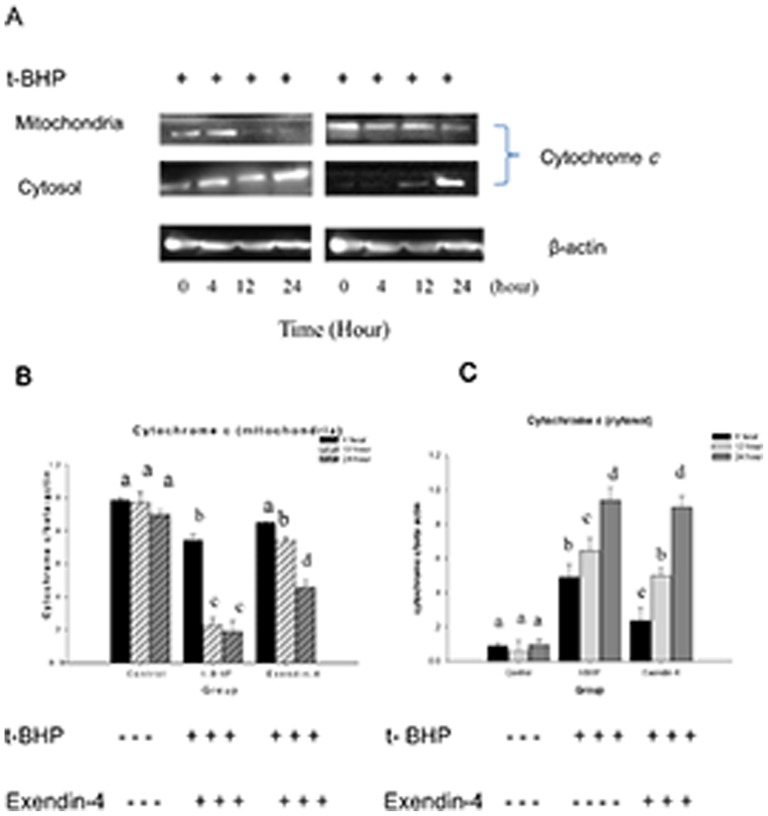
Exendin-4 attenuates the release of cytochrome *c* from the Min6 cell mitochondria. Figure A shows the effect of Exendin-4 on cytochrome *c* in Min6 cells. The cells were treated with or without Exendin-4 and then with t-BHP for different intervention times. The cells were then collected, and cytoplasmic mitochondrial fractions were prepared using a mitochondrial/cytosol fraction kit. The samples from each fraction were analyzed using Western blot for cytochrome *c*. Figure B presents the mitochondria. Figure C includes the group gray comparison (cytoplasm). The data are expressed as the means±S.D. (*n* = 6). a, b, c, d and e indicate the blank comparison and the t-BHP (200 µM for 24 hours) and Exendin-4 (100 µM for 48 hours) group comparisons (*p*<0.05).

**Figure 9:**
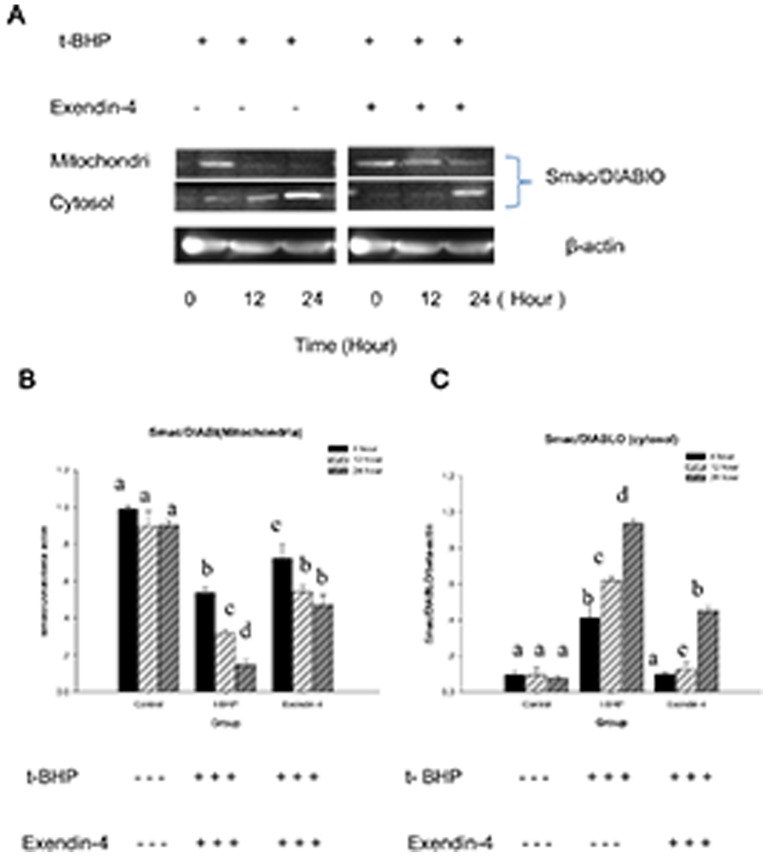
Exendin-4 attenuates the release of Smac/DLAMO from the Min6 cell mitochondria. Figure A shows the effect of Exendin-4 on Smac/DLAMO in Min6 cells. The cells were treated with or without Exendin-4 and then with t-BHP for different intervention times. The cells were then collected, and cytoplasmic mitochondrial fractions were prepared with a mitochondrial/cytosol fraction kit. The samples from each fraction were analyzed using Western blot for Smac/DLAMO. Figure B presents the mitochondria. Figure C includes the group gray comparison (cytoplasm). The data are expressed as the means±S.D. (*n* = 6). a, b, c, d and e indicate the blank comparison and the t-BHP (200 µM for 24 hours) and Exendin-4 (100 µM for 48 hours) group comparisons (*p*<0.05).

Because Exendin-4 can protect mitochondrial function from ROS-inducing treatment, we examined whether it also prevent the release of cytochrome *c*. Western blot results showed that compared with the control, Exendin-4 (100 µM for 48 hours) reduced the expression of cytochrome *c* in the cytoplasm (*n* = 6; *p*<0.05; Figure 8). We also examined the mitochondrial protein Smac/DLAMO, which promotes cytochrome *c*-dependent caspase activation by eliminating IAP inhibition. We found that compared to the control group, Exendin-4 also attenuates the t-BHP-induced Smac/DLAMO release from the mitochondria (*n* = 6; *p*<0.05; Figure 9).

### Exendin-4 (100 µM for 48 hours) reduces caspase 3 activity

**Figure 10:**
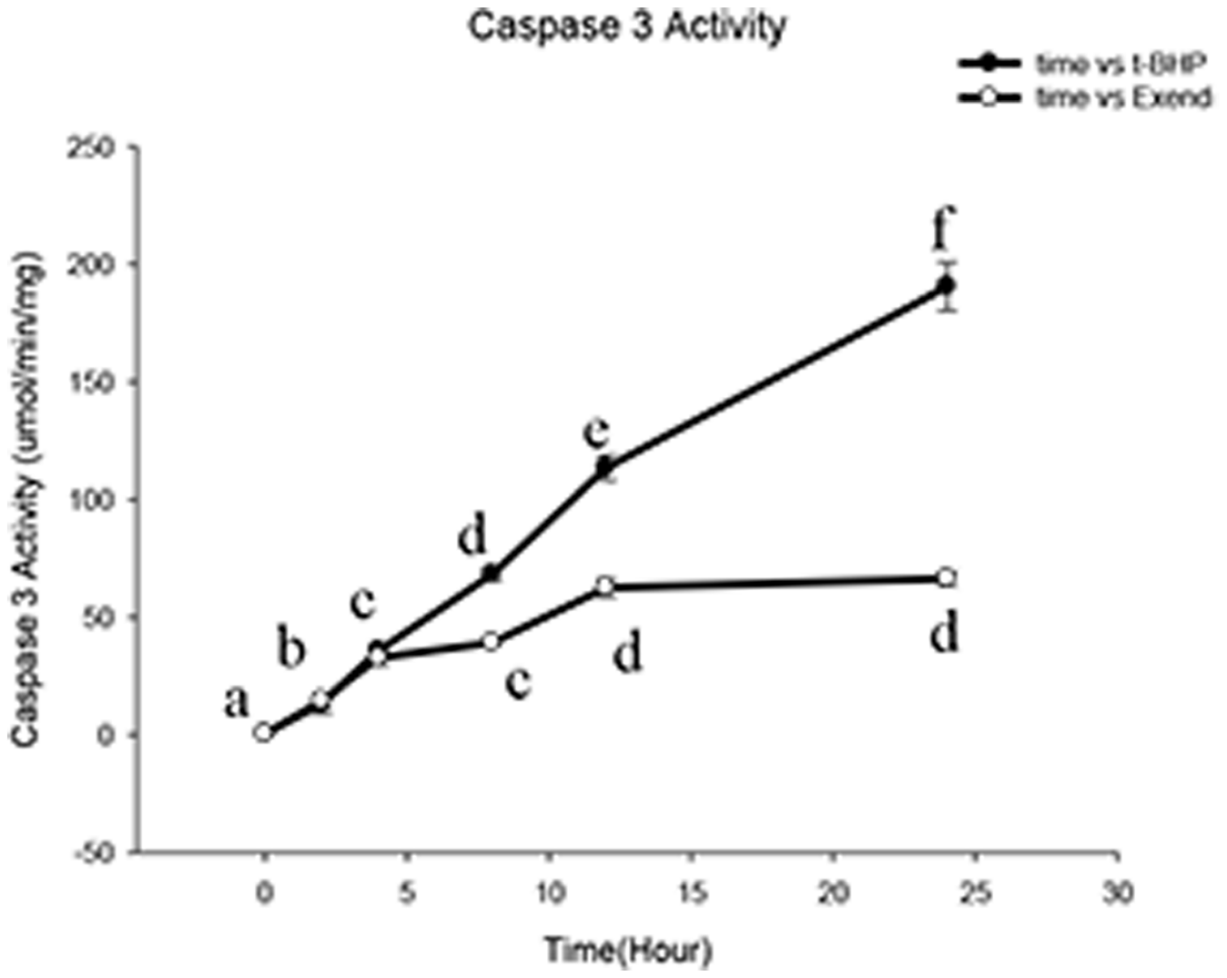
Exendin-4 reduces the Min6 cell apoptosis factor caspase 3 activity. This figure presents a comparison of the caspase 3 activity in the t-BHP group and the Exendin-4 group. Caspase 3 activity was determined with a caspase 3 assay kit. The data are expressed as the means±S.D. (*n* = 6). a, b, c, d, e and f indicate comparisons of the two groups (*p*<0.05).

The caspase 3 activity was measured using a caspase 3 activity assay kit. The results showed that as the reaction time increased at a t-BHP concentration of 200 µM, the caspase 3 expression level progressively increased. Incubation with Exendin-4 for 48 hours attenuated the accelerating increase in the expression level of caspase 3 and decreased its production. There was a statistically significant difference between the two groups (*n* = 6; *p*<0.05; Figure 10).

**Figure 11:**
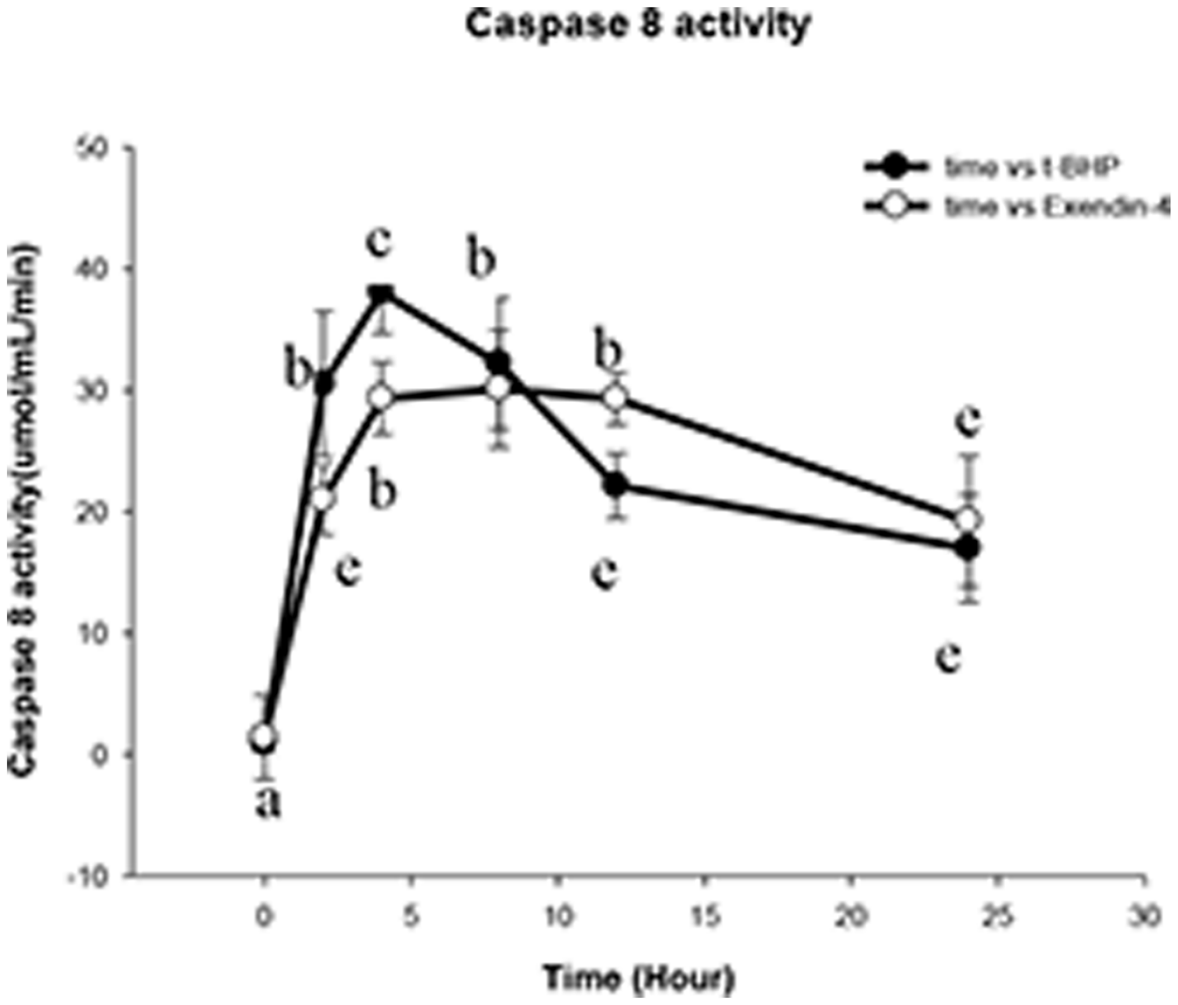
Exendin-4 cannot reduce the Min6 cell apoptosis factor caspase 8 activity. This figure presents a comparison of the caspase 8 activity of the t-BHP group and the Exendin-4 group. Caspase 8 activity was determined using a caspase 8 assay kit. The data are expressed as the means±S.D. (*n* = 6). a, b, c, d and e indicate comparisons between the two groups (*p*<0.05).

The caspase 8 activity was measured using a caspase 8 activity assay kit. The results showed that as the reaction time increased at a t-BHP concentration of 200 µM, the quantity of caspase 8 expression progressively increased; however, compared with the control group, there was no statistically significant difference between the control and Exendin-4 (100 µM for 48 hours) groups (*p*>0.05; Figure 11).

### No significant correlation between cPLA2 and ROS-induced Min6 cell apoptosis

**Figure 12:**
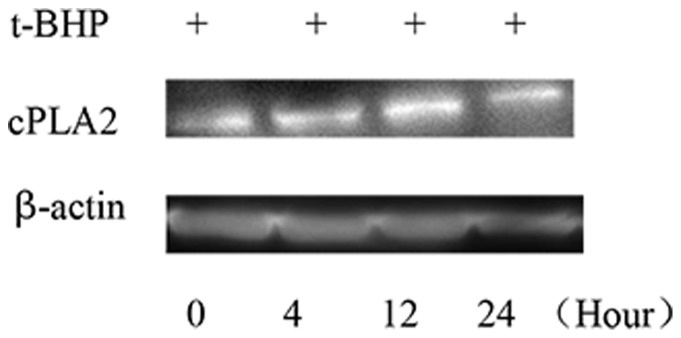
cPLA2 may not participate in t-BHP-induced apoptosis. Western blot analysis of cPLA2 expression during t-BHP-induced apoptosis. The cells were treated with or without Exendin-4 and then with t-BHP (200 µM for 24 hours) for different intervention times. The cells were then collected, and cell lysates were prepared and analyzed for cPLA2 and actin.

In this study, we did not observe any direct relationship between cPLA2 expression and cell apoptosis. For the t-BHP 200 µM 24-hour group, we did not observe any relationship between cPLA2 expression and Min6 cell apoptosis (Figure 12).

### iPLA2 was transcriptionally downregulated in t-BHP-induced apoptosis, and Exendin-4 (100 µM 48 hour) is positively correlated with iPLA2 transcription

Exendin-4 plays an important role in the protection of mitochondria during t-BHP-induced apoptosis, and under physiological conditions, mitochondria can repair peroxidative damage in part through a remodeling mechanism via the deacylation-reacylation cycle mediated by phospholipase A_2_. Consequently, we investigated whether iPLA2 plays a role in protecting mitochondrial function from damage caused by mitochondrial-generated ROS during apoptotic induction by t-BHP and whether the Exendin-4 protective function is related to iPLA2.

**Figure 13:**
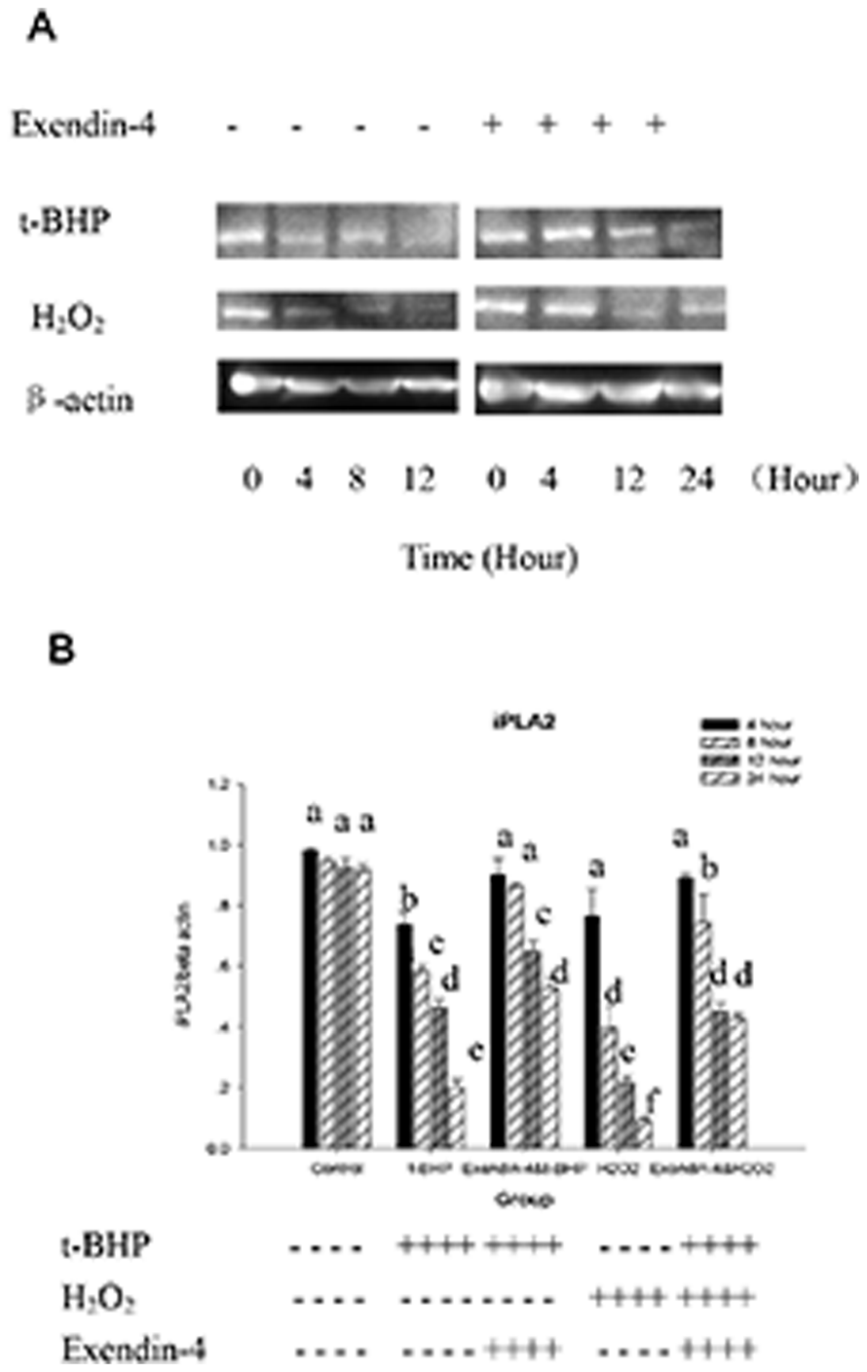
iPLA2 expression is downregulated in t-BHP-induced apoptosis. Figure A, Western blot analysis of iPLA2 expression during t-BHP-induced apoptosis. The cells were first treated with or without Exendin-4 and then with t-BHP (200 µM for 24 hours) or H_2_O_2_ (100 µM for 12 hours) for different intervention times. The cells were then collected, and cell lysates were prepared and analyzed for iPLA2 and actin. B, the group gray comparison. Data are expressed as the means±S.D. (*n* = 6). a, b, c, d, e and f indicate group comparisons (p<0.05).

**Figure 14:**
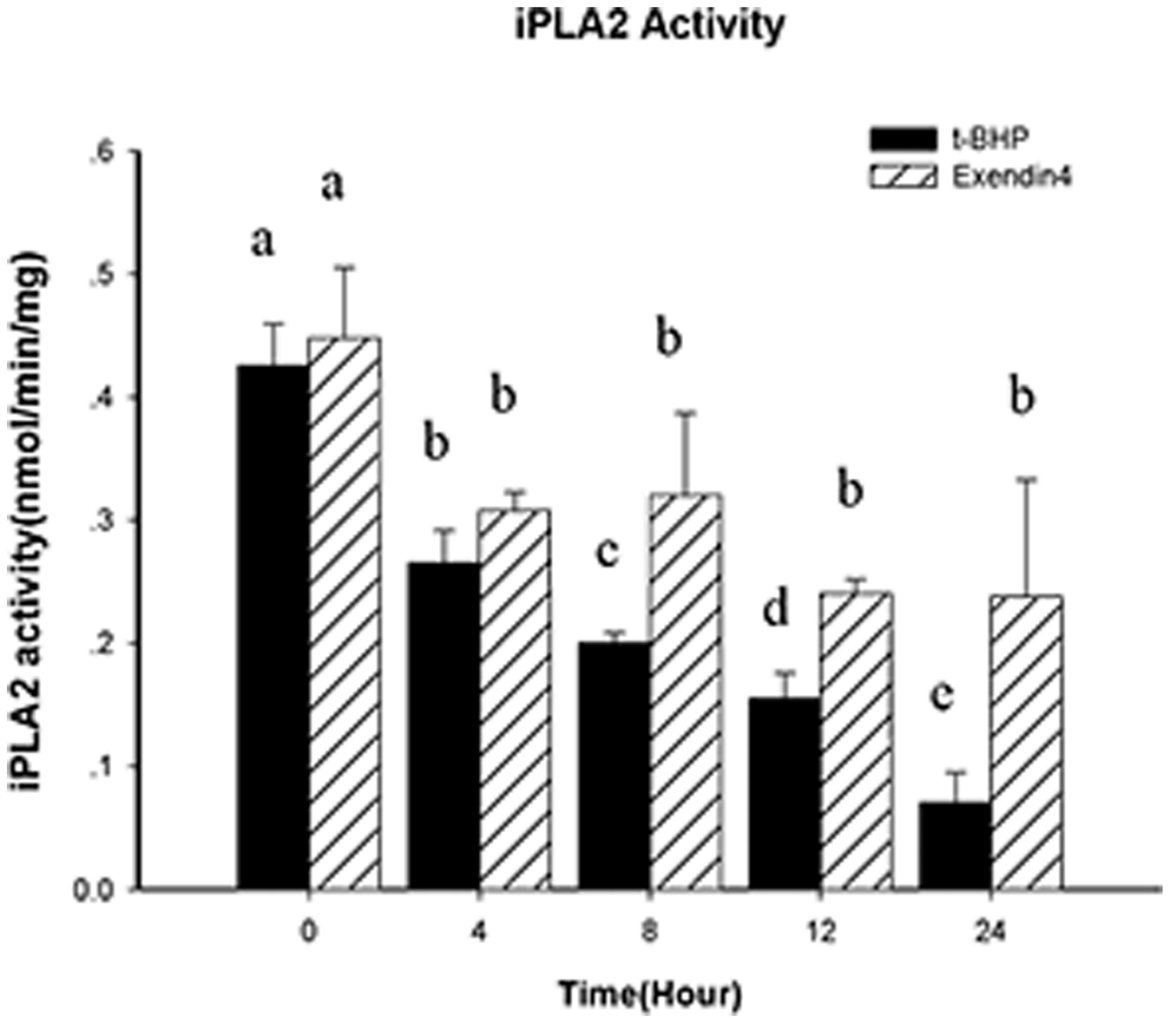
iPLA2 activity is downregulated in t-BHP-induced apoptosis. Cells were first treated with or without Exendin-4 and then with t-BHP (200 µM for 24 hours) for different intervention times. The cells were then collected, and cell lysates were prepared and analyzed for iPLA2 activity. Data are expressed as the means±S.D. (*n* = 6). a, b, c, d and e indicate group comparisons (p<0.05).

We found that compared with the t-BHP (200 µmol/l for 24 hours) group, the group that was treated with Exendin-4 (100 µM for 48 hours) had higher t-BHP-induced iPLA2 expression (*p*<0.05). The magnitude of the decrease in the time-concentration curve was lower in the Exendin-4 (100 µM for 48 hours) group, and the changes were not significant after 8 hours. We also examined the H_2_O_2_ (50 µM for 24 hours) group and found that after treatment with Exendin-4 (100 µM for 48 hours), the H_2_O_2_-induced iPLA2 expression exhibited the same trend (*p*<0.05; Figure 13). iPLA2's activity was correlated with its protein levels (Figure 14).

**Figure 15:**
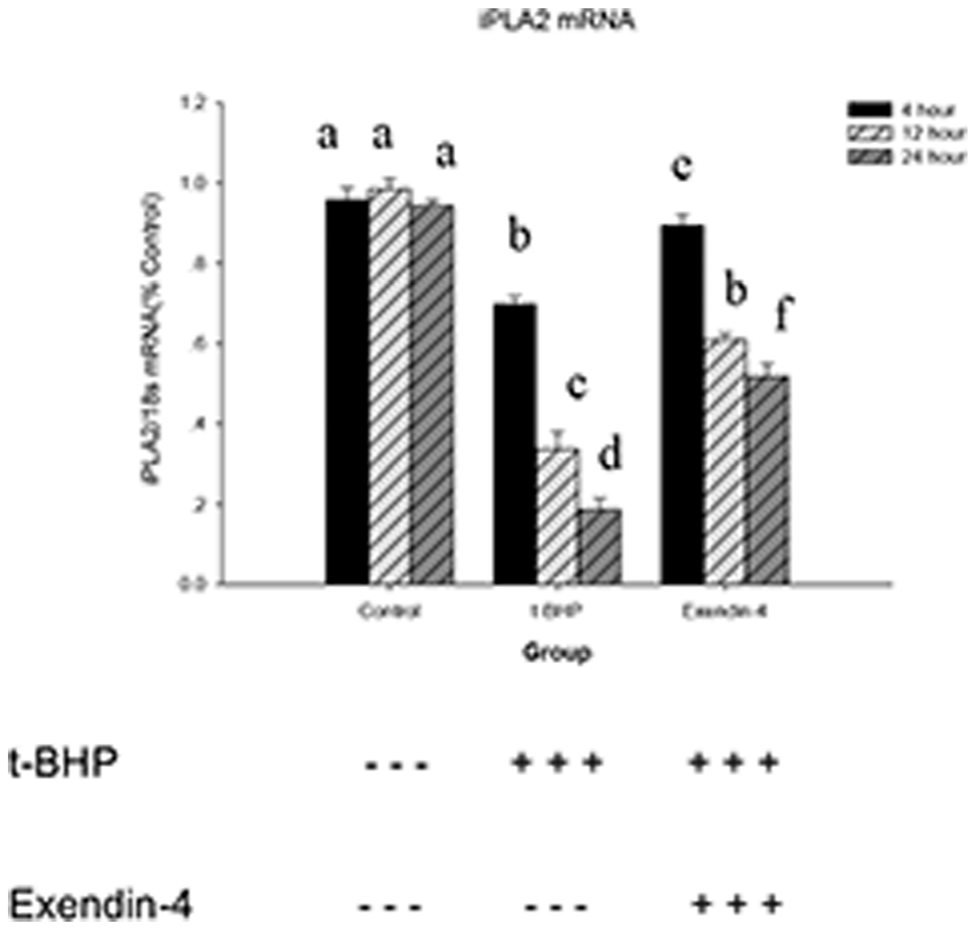
iPLA2 mRNA expression analysis. This figure presents the RT-PCR analysis of iPLA2 mRNA during t-BHP treatment with or without prior Exendin-4 treatment. Total RNA was isolated from each sample, and aliquots were subjected to RT-PCR analysis for iPLA2 and 18S. The data are expressed as the means±S.D. (*n* = 6). a, b, c, d, e and f indicate group comparisons of the control, t-BHP and Exendin-4 groups (*p*<0.05).

To confirm that t-BHP downregulates iPLA2 transcription, we used RT-PCR to quantify iPLA2 mRNA levels in the t-BHP group and the Exendin-4 group (after treatment with Exendin-4 100 µM for 48 hours, the cells were treated with 100 µM t-BHP for 24 hours). Our results suggest that t-BHP not only induces the oxidative stress that leads to the peroxidation of mitochondrial membrane phospholipids but also impairs the repair system by downregulating iPLA2 transcription. Exendin-4 can attenuate this condition (Figure 15).

## Discussion

Numerous types of chronic damage, including glucotoxicity, lipotoxicity and amylin, are important mechanisms for the progression of type 2 diabetes mellitus (T2DM). Eventually, these types of damage lead to increased pancreatic β-cell mitochondrial ROS production, mitochondrial inner membrane cardiolipin oxidative damage and pancreatic β-cell apoptosis. Previous research has shown that Exendin-4 reduces pancreatic β-cell apoptosis at the gene level, promotes pancreatic β-cell proliferation and differentiation, improves insulin resistance through numerous mechanisms, reduces metabolic damage and improves the T2DM pathological status [23]–[28].

Based on the literature published in China and overseas, we chose superoxide t-BHP and H_2_O_2_ to establish a Min6 cell oxidative damage model. The results showed that as the rate of cell apoptosis increased, the intracellular oxidative lipid content increased, which revealed that oxidative stress was the main mechanism of mitochondrial damage in Min6 cells in this study. Superoxide t-BHP- and H_2_O_2_-induced Min6 cell apoptosis showed time-concentration dependency, with two peak values at t-BHP (200 µM for 1 hour) and t-BHP (200 µM for 24 hours). This result may be related to the acute chemical injury induced by the initial effect of a strong oxidizing agent and the initiation of the cells' self-protective stress response. In the t-BHP (200 µM for 24 hours) group, the Min6 cell mitochondrial damage index (membrane potential and ROS) increased. The expression of caspase 3 and 8 apoptosis factors increased, and mitochondrial cytochrome c and apoptosis protein Smac/DLAMO were released into the cytoplasm. The results suggest that oxidative damage plays an important role in the process of pancreatic β-cell apoptosis.

The group treated with Exendin-4 (100 µM for 48 hours) showed reductions in the rate of cell apoptosis, mitochondrial ROS production, membrane potential, production of apoptosis factor caspase 3, release of mitochondrial protein cytochrome c and apoptosis protein Smac/DLAMO into the cytoplasm, and oxidative damage, compared with the t-BHP (200 µM for 1 hour) group. These results suggest that Exendin-4 has a protective role in mitochondrial function and can reduce pancreatic β-cell apoptosis.

iPLA2 is an important phospholipid remodeling and repair factor that enriches the inner mitochondrial membrane cardiolipin in polyunsaturated fatty acids through deacylation and acylation. iPLA2 allows the inner mitochondrial membrane to maintain structural completeness and normal function and makes important contributions to oxidative damage resistance. Previous research has shown that iPLA2 plays a key regulatory role in oxidative stress-induced pancreatic cell apoptosis and is closely related to the programed apoptosis of pancreatic cells [29]–[35]. In this study, before and after the addition of Exendin-4 (100 µM for 48 hours), the Min6 cell apoptosis rate was observed to be positively related to iPLA2 expression; apoptosis was reduced in Min6 cells with high levels of iPLA2 expression, and this tendency was related to Exendin-4.

Much research has been published on the anti-apoptotic mechanism of Exendin-4 in the pancreatic β-cell. The research has focused on the cAMP activation of phosphatidylinositol 3-kinase (PI3K) and its downstream mitogen-activated protein kinase (MAPK/ERK) and protein kinase B (PKB/Akt) and protein kinase A (PKA) signal transduction pathways; the regulation of caspase 3, Fas and apoptosis promoting genes, such as programmed cell apoptosis factor 5 (PDCD25); the regulation of the expression of anti-apoptotic proteins, such as Bcl-2 and Bcl-xL; the enhancement of PI3K and PKC activity; the induction of pancreatic and duodenal homeobox-1 (PDX-1) gene expression; and the induction of early cell differentiation-related genes, such as C-fox, C-jun, Jun-B, Zif-268 and Nur-77, to reduce pancreatic β-cell apoptosis. However, the exact mechanism of Exendin-4 in pancreatic β-cells remains unknown. This study is the first to use an Exendin-4 agent synthesized through biofermentation technology (the pET32a(+) plasmid and the synthetic Exendin-4 gene fragment were digested with KpnI and HindIII, and they were ligated together using a ligase to form the recombinant plasmid. The plasmid was then transformed into the BL21(DE3) E. coli strain to obtain the recombinant genetic engineering strain. After high-density fermentation, chromatography, enzyme digestion and repeated chromatography, the stock solution was obtained). Additionally, it is the first to use oxidative stress as the breakthrough point for preliminary investigations of the relationship between iPLA2's anti-oxidative mechanism and the anti-apoptotic role of Exendin-4 in pancreatic β-cells. We showed that Exendin-4 could reduce pancreatic β-cell apoptosis and mitochondrial oxidative stress and that these roles may be related to iPLA2. A direct correlation requires further research.
